# Chloroquine exposure triggers distinct cellular responses in sensitive versus resistant *Plasmodium falciparum* parasites

**DOI:** 10.1038/s41598-018-29422-6

**Published:** 2018-07-24

**Authors:** Sarah J. Reiling, Georg Krohne, Oliver Friedrich, Timothy G. Geary, Petra Rohrbach

**Affiliations:** 1 Institute of Parasitology, McGill University, Ste. Anne de Bellevue (Montréal), Québec, Canada; 2 Theodor Boveri Institute, Biocenter, University of Würzburg, Würzburg, Germany; 3 Institute of Medical Biotechnology, Friedrich-Alexander University of Erlangen-Nürnberg, Erlangen, Germany

## Abstract

Chloroquine (CQ) treatment failure in *Plasmodium falciparum* parasites has been documented for decades, but the pharmacological explanation of this phenotype is not fully understood. Current concepts attribute CQ resistance to reduced accumulation of the drug at a given external CQ concentration ([CQ]_ex_) in resistant compared to sensitive parasites. The implication of this explanation is that the mechanisms of CQ-induced toxicity in resistant and sensitive strains are similar once lethal internal concentrations have been reached. To test this hypothesis, we investigated the mechanism of CQ-induced toxicity in CQ-sensitive (CQS) *versus* CQ-resistant (CQR) parasites by analyzing the time-course of cellular responses in these strains after exposure to varying [CQ]_ex_ as determined in 72 h toxicity assays. Parasite killing was delayed in CQR parasites for up to 10 h compared to CQS parasites when exposed to equipotent [CQ]_ex_. In striking contrast, brief exposure (1 h) to lethal [CQ]_ex_ in CQS but not CQR parasites caused the appearance of hitherto undescribed hemozoin (Hz)-containing compartments in the parasite cytosol. Hz-containing compartments were very rarely observed in CQR parasites even after CQ exposures sufficient to cause irreversible cell death. These findings challenge current concepts that CQ killing of malaria parasites is solely concentration-dependent, and instead suggest that CQS and CQR strains fundamentally differ in the consequences of CQ exposure.

## Introduction

Although extensive malaria control measures have significantly decreased the incidence of malaria worldwide^1^, antimalarial drug resistance remains a serious concern. Much remains unknown about drug resistance mechanisms, hampering strategies for disease control or eradication. It is imperative to understand the mechanisms of drug resistance to currently used antimalarials as new chemotherapeutic approaches are pursued.

Chloroquine (CQ), a 4-aminoquinoline derivative, has been a highly efficacious, safe and low-cost antimalarial drug. Resistance to this drug spread worldwide and required decades to evolve^2^, but has now dramatically limited its efficacy against *Plasmodium falciparum*, the most lethal species of human malaria parasites. CQ diffuses through biological membranes in its uncharged form and accumulates, due to its weak base properties, in the parasite’s acidic digestive vacuole (DV)^3^. The main function of the DV is the proteolysis of hemoglobin, a process that generates dipeptides and ferriprotoporphyrin IX (FPIX)^4^. High concentrations of FPIX are potentially toxic to the parasite because FPIX permeabilizes membranes, leading to cell lysis^5,6^. To overcome this, the parasite biocrystallizes FPIX dimers into inert hemozoin (Hz) crystals^7^, a process that is inhibited by CQ and is thought to result in increased levels of toxic FPIX^8–11^. Several processes are impaired by high concentrations of FPIX. The primary effect of the CQ-FPIX complex is most likely membrane alteration, which interferes with the docking of mature hemoglobin-laden endocytic vesicles to the DV membrane^12^. Furthermore, CQ-FPIX complexes are hypothesized to lead to the permeabilization of the DV membrane, resulting in the release of DV contents into the cytosol^5,13^ and causing irreversible damage to the parasite.

Resistance to CQ has primarily been attributed to a point mutation in the *P*. *falciparum* chloroquine resistance transporter (PfCRT) at amino acid position 76^14,15^. PfCRT harboring the K76T mutation is proposed to transport CQ out of the DV away from its primary site of action^16^, thereby limiting the vacuolar concentration of CQ available to bind to FPIX and reducing cell damage. A corollary to this hypothesis is that CQ-resistant (CQR) strains of *P*. *falciparum* should be killed once CQ concentrations in the DV ([CQ]_DV_) that kill CQ-sensitive (CQS) are reached, an equilibrium that requires higher external [CQ]. However, CQR parasites are reported to tolerate higher CQ levels in the DV than CQS strains, an observation that cannot be explained by simple concentration-dependent toxicity effects of CQ. If CQS and CQR parasites are exposed to concentrations of extracellular chloroquine ([CQ]_ex_) that generate equal internal CQ-concentrations in the DV ([CQ]_DV_), equipotent CQ-dependent killing should be observed. However, CQR parasites have higher survival rates than CQS parasites at the same [CQ]_DV_^17^. It appears that the PfCRT K76T mutation alone cannot fully explain resistance to CQ from stoichiometric considerations only.

In this study, we report differences in killing kinetics and cell biological consequences in CQS and CQR parasites after exposure to equipotent [CQ]_ex_, determined based on IC_50_ values in assays quantifying growth inhibition. Our findings show that CQS and CQR parasites display distinct cellular responses as a consequence of CQ exposure. These results suggest that our understanding of the mechanism of action of CQ requires refinement, and that cell biological effects of CQ on sensitive and resistant parasites need to be investigated in parallel.

## Results

### *In situ* sensitivity of CQ

Since measurements of drug responses can vary among laboratories, partly due to different culture conditions and procedures, we first determined CQ IC_50_ values for the strains used in this study in 72 h SYBR Green I viability assays (Table 1), as described previously^18,19^. IC_50_ values and their respective SEMs for the CQS parasite strains 3D7 and HB3 were 24 ± 6 nM and 14 ± 1 nM, respectively, and 169 ± 4 nM and 166 ± 9 nM, respectively, for the CQR parasite strains Dd2 and FCB. Resistance to CQ, as indicated by IC_50_ values, was partially reversed in CQR strains by co-incubation with 1 µM verapamil (VP), while no significant difference in IC_50_ values was observed with VP in CQS parasite strains (p > 0.05), as expected. All IC_50_ values are in good agreement with those obtained by other groups^19–23^. Subsequent experiments with CQ were based on these IC_50_ values.

**Table 1:** IC_50_ values and PfCRT and PfMDR1 mutations of *P*. *falciparum* parasites used in this study.

Strain	CQ	CQ + VP	VP	PfCRT	PfMDR1				
	(nM)	(nM)	(µM)	76	86	184	1034	1042	1246
3D7	24 ± 6	17 ± 1	38 ± 6	K	N	Y	S	N	D
HB3	14 ± 1	19 ± 2	50 ± 2	K	N	F	S	D	D
Dd2	169 ± 4	53 ± 7	33 ± 4	T	Y/F^a^	Y	S	N	D
FCB	166 ± 9	41 ± 7	34 ± 7	T	Y	Y	S	N	D

Values obtained from three independent experiments ± SEM.

CQ, chloroquine; VP, verapamil.

^a^Described in^26,27^.

### Onset of growth inhibition is delayed in CQR parasites

**Figure 1:**
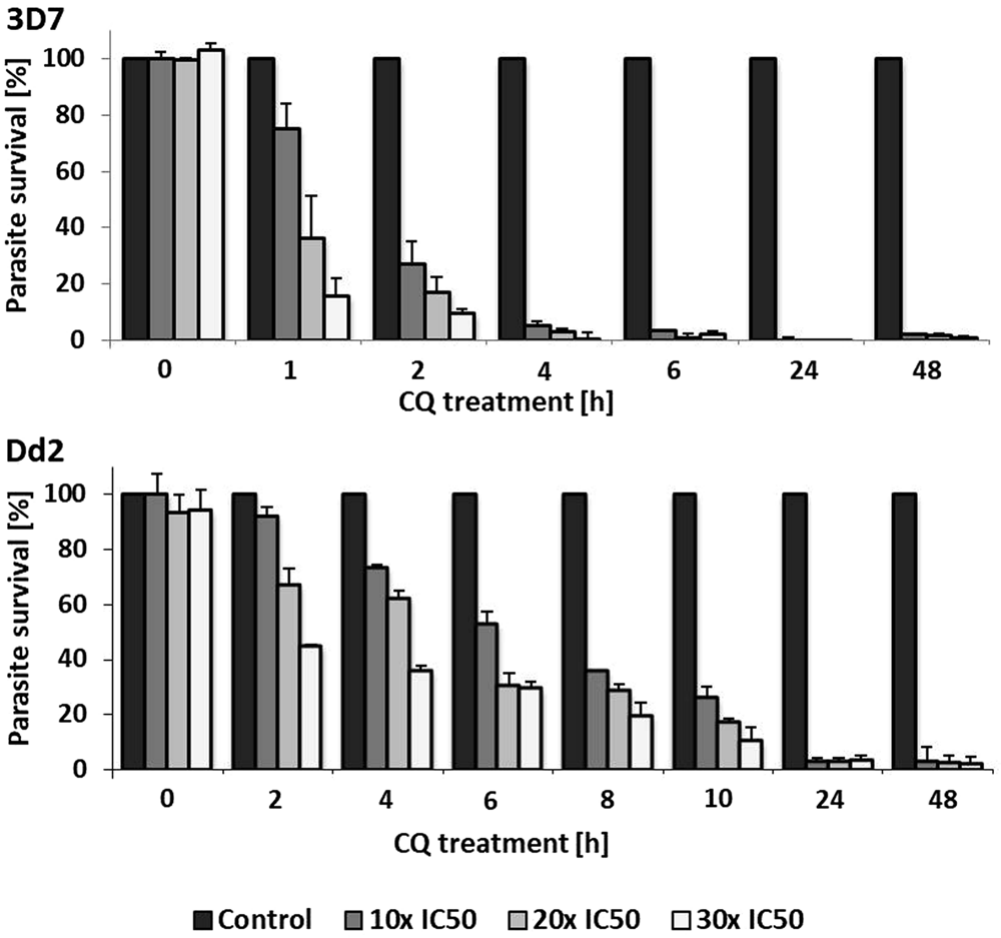
Determining parasite growth inhibition in CQS and CQR parasites. 3D7 and Dd2 parasites were exposed to their respective 10x, 20x or 30x IC_50_ CQ values for the time indicated, or left untreated (control). Analysis was performed using the SYBR Green I detection assay. Percent growth relative to control was calculated for each time point. Results shown are from at least three independent experiments. Error bars represent SEM.

The time course of CQ-induced growth inhibition of CQS and CQR parasites was analyzed using equipotent [CQ]_ex_ as determined above. CQS 3D7 and CQR Dd2 parasite-infected RBCs (iRBCs) were exposed to [CQ]_ex_ equal to 10x, 20x and 30x their respective IC_50_ values (to provide equivalently lethal exposures for CQS and CQR parasites) or left untreated (Fig. 1). To confirm that the wash step was sufficient to remove CQ from the culture medium, parasites from the 0 h treatment groups were incubated with the appropriate [CQ]_ex_ and washed immediately. No changes in parasite survival relative to the untreated control group were observed (Fig. 1). Parasite survival was decreased by exposure to all [CQ]_ex_ at all other time points; killing of CQS 3D7 parasites occurred more rapidly than killing of CQR Dd2 parasites at equally toxic [CQ]_ex_. The responses to CQ exposure in 3D7 and Dd2 parasites started to diverge after 2 h. After 4 h of CQ exposure, Dd2 parasite survival rates were 73% for 10x IC_50_ [CQ]_ex_, 62% for 20x IC_50_ [CQ]_ex_, and 36% for 30x IC_50_ [CQ]_ex._ In comparison, a marked decrease in growth (<10% survival) was observed in 3D7 parasites for all [CQ]_ex_. For Dd2 parasites, an equivalent level of growth inhibition (<10% survival) was observed only after >10 h CQ exposure. These results show that longer exposure of CQR parasites to equipotent [CQ]_ex_ is required for growth inhibition compared to CQS parasites.

### The digestive vacuolar membranes of CQS and CQR parasites remain intact

Disruption of the DV membrane and leakage of DV contents into the parasite cytosol have been hypothesized to be the main contributors to parasite death after CQ exposure^24^. We evaluated leakage of DV contents into the parasite cytosol after exposure to regimens of [CQ]_ex_ that elicited irreversibly lethal damage. Prior to CQ exposure, 3D7 and Dd2 parasites were loaded with Fluo-4 AM, a fluorophore that accumulates in the DV and remains trapped within this compartment^25–27^.

**Figure 2:**
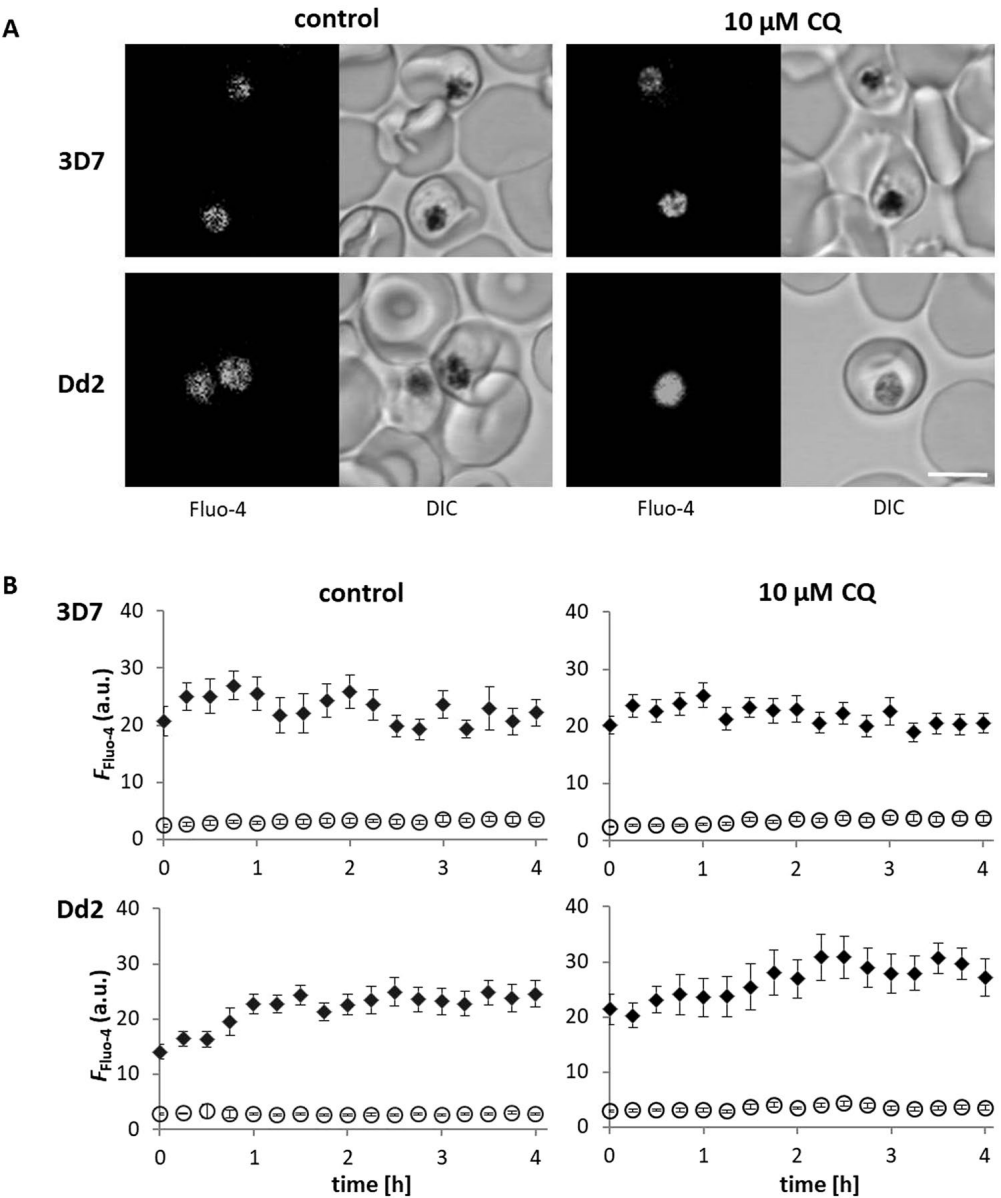
Analysis of DV membrane integrity. 3D7 and Dd2 parasites were pre-incubated with 5 µM Fluo-4 AM in Ringer’s solution for 50 min and then placed in Ringer’s solution only (control) or Ringer’s solution containing 10 µM CQ. The same parasites were imaged in 15 min intervals. (**A**) Fluo-4 fluorescence and DIC images of representative parasites are shown after 4 h CQ exposure. Scale bar, 5 µm. (**B**) relative fluorescence intensity of Fluo-4 in the DV (black diamonds) or cytosol (open circles) of the parasites was determined. The ratio was calculated relative to background fluorescence. Results shown are from three independent experiments performed on separate days; more than 28 parasites were evaluated. Error bars represent SEM.

Fluo-4 AM loaded 3D7 and Dd2 parasites were exposed for up to 4 h to 10 μM CQ, which corresponds to 400x and 60x their IC_50_, respectively, as derived from the IC_50_ concentrations (Fig. 1). Changes in Fluo-4 fluorescence were monitored in cytosol and DV of control and CQ-exposed CQS and CQR parasites using confocal microscopy. Strong DV and minimal cytosolic Fluo-4 fluorescence was observed in all parasites and remained stable throughout the 4 h time period. Redistribution of Fluo-4 fluorescence from the DV to the cytosol was not detected, suggesting that the DV membrane remained intact in control and CQ-exposed parasites (Fig. 2). The slight increase in DV Fluo-4 fluorescence seen over time in control and CQ-exposed Dd2 parasites (Fig. 2B) is likely due to the known transport of Fluo-4 into the DV of this parasite^27^.

### Effect of CQ on parasite morphology

Hz crystals are formed and stored in the DV. Hz crystals are easily recognized as dark structures under light microscopy. Live cell imaging reveals that Hz crystals move freely within the DV in an apparently random manner. We observed that Hz crystals appear smaller and move more rapidly in CQR strains (Dd2, FCB, FCR3, K1) compared to CQS strains (3D7, HB3, NF45, D10) (data not shown). Accordingly, exposure of CQR and CQS strains to CQ in culture may affect the movement and localization of Hz within these parasites differently.

To test this hypothesis, synchronized mid-trophozoite stage parasites (CQS strains: 3D7 and HB3; CQR strains: Dd2 and FCB) exposed to equitoxic [CQ]_ex_ were monitored for 4 h using differential interference contrast (DIC) microscopy. The characteristic rapid movement of Hz crystals in CQR strains was not altered in Dd2 (Supplementary Videos S1 and S2) and FCB parasites, in which Hz movement persisted even after 24 h CQ exposure. In contrast, Hz movement was already markedly reduced in the CQS strains 3D7 (Supplementary Videos S3 and S4) and HB3 after 4 h exposure to CQ.

**Figure 3:**
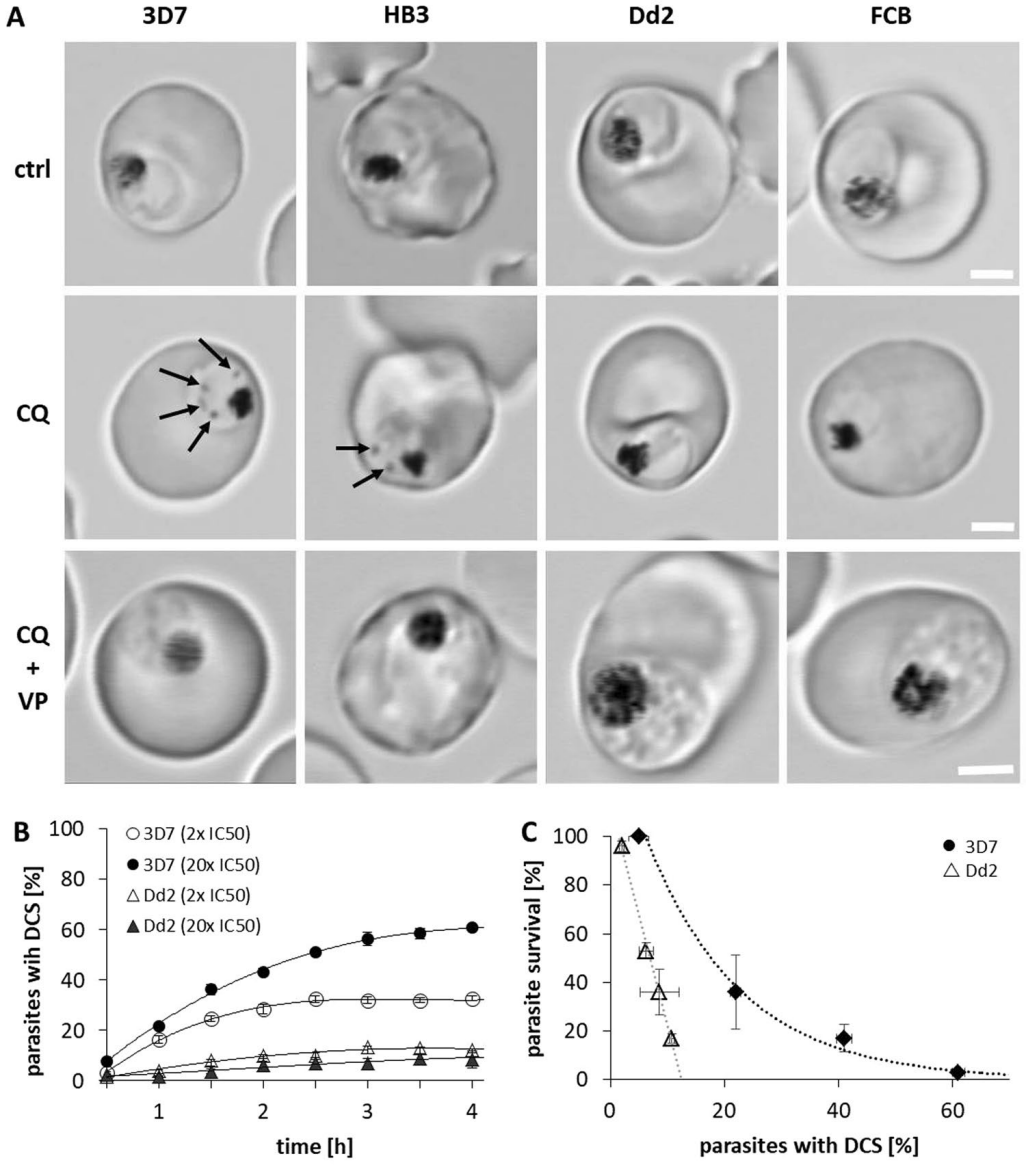
Detection of DCS after CQ treatment. DCS appeared in the cytosol of CQS parasites after CQ exposure. These structures were scarce in CQR parasites, even after high [CQ] exposure. (**A**), CQS (3D7, HB3) and CQR (Dd2, FCB) strains were incubated with 500 nM CQ, 500 nM CQ + 10 µM VP, or Ringer’s solution only (ctrl) for 2–3 h. DCS (arrows) were only found in the cytosol of CQS parasites treated with CQ. Scale bar, 2 µm. (**B**) 3D7 and Dd2 parasites were incubated with various [CQ] and the percentage of parasites with DCS was determined at 30 min intervals. Results are from three independent experiments; more than 3,000 parasites were evaluated. Error bars represent SEM. (**C**) correlation between parasite survival and number of 3D7 (black circles) and Dd2 parasites (open triangles) containing DCS at the respective survival rates. Results are from three independent experiments. Error bars represent SEM.

In addition to reduced Hz movement, we also observed the appearance of dark, rapidly moving structures in the cytosol of CQS parasites within 1 h of CQ exposure (Fig. 3A). These compartments appeared to be moving freely and randomly (Supplementary Video S4). The number of CQS parasites containing these dark cytosolic structures (DCS) steadily increased over time. In contrast, very few CQR parasites were observed to have DCS (<10% after 4 h CQ exposure), even at 30x the IC_50_ value of CQ (Fig. 3A; Supplementary Figure S1; Supplementary Video S2). DCS were readily visible when CQ-exposed CQS parasites were methanol-fixed but not Giemsa-stained (Supplementary Figure S2).

Verapamil (VP) has been shown to reverse CQ drug resistance in CQR parasites. To verify if VP affects the appearance of DCS in CQ treated parasites, 10 µM VP was added to CQS and CQR parasites treated with CQ. The addition of VP suppressed the appearance of DCS in CQS parasites and had no effect on CQR parasites (Fig. 3A).

To determine the rate of appearance of DCS in CQS and CQR parasites, 3D7 and Dd2 strains were exposed to 20x their IC_50_ [CQ]_ex_ values for up to 4 h. The number of DCS steadily increased in 3D7 parasites within the first 1–4 h (22%, 43%, 56%, and 61%, respectively) until a plateau was reached (Fig. 3B). DCS still appeared in 3D7 parasites at [CQ]_ex_ = 2x the IC_50_ value, but the number of parasites containing DCS was reduced (33% versus 61% after 4 h). In contrast, very few CQ-exposed Dd2 parasites had detectable DCS, even when exposed to 30x the IC_50_ [CQ]_ex_ value (2%, 6%, 7% and 9% after 1–4 h, respectively; Fig. 3B; Supplementary Figure S1). These values did not change substantially when Dd2 parasites were exposed to 2x their IC_50_ [CQ]_ex_ value.

The appearance of DCS was linked with growth inhibition in CQS 3D7 parasites compared to CQR Dd2 parasites (Fig. 3C). These findings support the hypothesis that exposure to equipotent [CQ] causes different cellular responses in CQS and CQR parasites.

### Ultrastructural analysis of CQ-exposed parasites

We employed TEM to better characterize the DCS. 3D7 and Dd2 parasites were exposed to CQ for 3 h at 20x their IC_50_ value, or left unexposed. To assure the structural preservation of *P*. *falciparum*-infected erythrocytes, parasites were enriched using a Percoll gradient. Non-enriched CQ-exposed and non-exposed parasites were also examined to confirm that Percoll enrichment did not affect parasite ultrastructure. No differences in the morphology of CQ-treated 3D7 parasites due to sample preparation were observed (Supplementary Figure S3).

**Figure 4:**
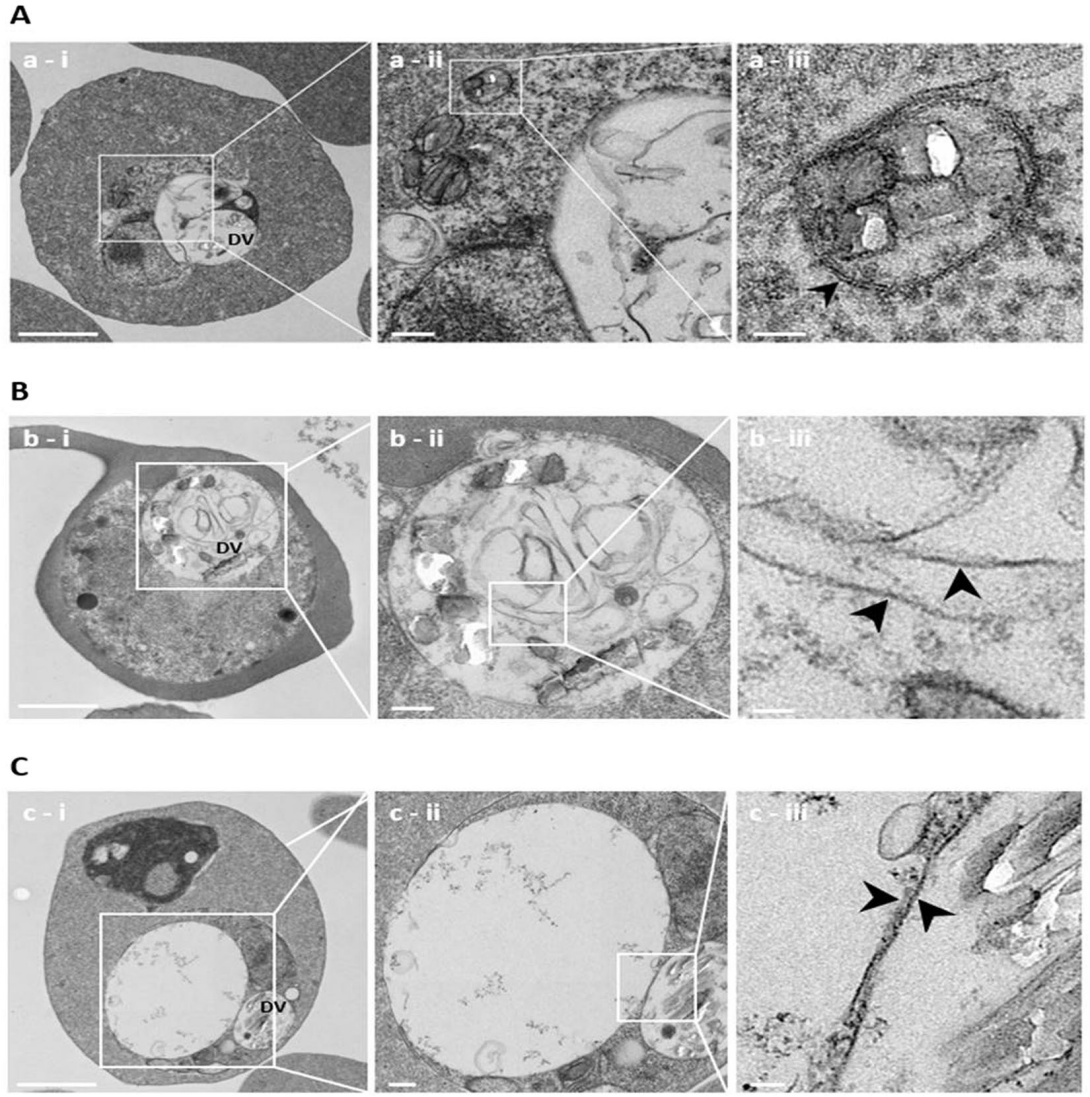
Electron microscopy images of CQ-exposed 3D7 parasites. 3D7 parasites were exposed to 20x IC_50_ CQ for 3 h, fixed and prepared for EM. (**A**), Hz crystals were detected in cytosolic compartments. Magnifications of the images show the intracellular localization of the Hz-containing structures and the surrounding membrane bilayer. (**B**) multiple undigested membranes are detected in the DV. (**C**) large vacuoles form in the parasite cytosol and exceed the size of the DV. Images were obtained at 15,000x magnification and digital zoom. Scale bars: i, 1 µm; ii, 200 nm; iii, 50 nm. Arrowheads: membrane bilayers. DV, digestive vacuole.

TEM images confirmed the presence of multiple DCS in 3D7 parasites (Fig. 4A). Since no three-dimensional images were taken and the individual TEM sections did not represent the entire parasite cytosol, the percentage of 3D7 parasites with DCS could not be determined in this experimental approach. DCS were surrounded by a membrane bilayer, were between 150–200 nm in diameter, and typically contained 1–5 Hz crystals. No Hz-containing compartments were observed in TEM images of CQ-treated Dd2 parasites (n = 17) or in 3D7 and Dd2 parasites that were not exposed to CQ (3D7, n = 17; Dd2, n = 29) (Supplementary Figure S4).

TEM images also revealed that CQ-exposed 3D7 parasites exhibited additional, previously undescribed, morphological changes after 3 h of 20x CQ IC_50_ exposure. In particular, abundant structures that appear to be membrane fragments are evident in the DV. Their lipid bilayer nature is readily visible (Fig. 4B). Furthermore, large vacuoles were present in the cytosol of CQ-exposed 3D7 parasites. These vacuoles were clearly distinct from, and were typically much larger than, the DV (Fig. 4C). These morphological changes were not seen in Dd2 parasites at that time point. No TEM slides were prepared for other time points.

### Hz-containing compartments likely originate from the DV

**Figure 5:**
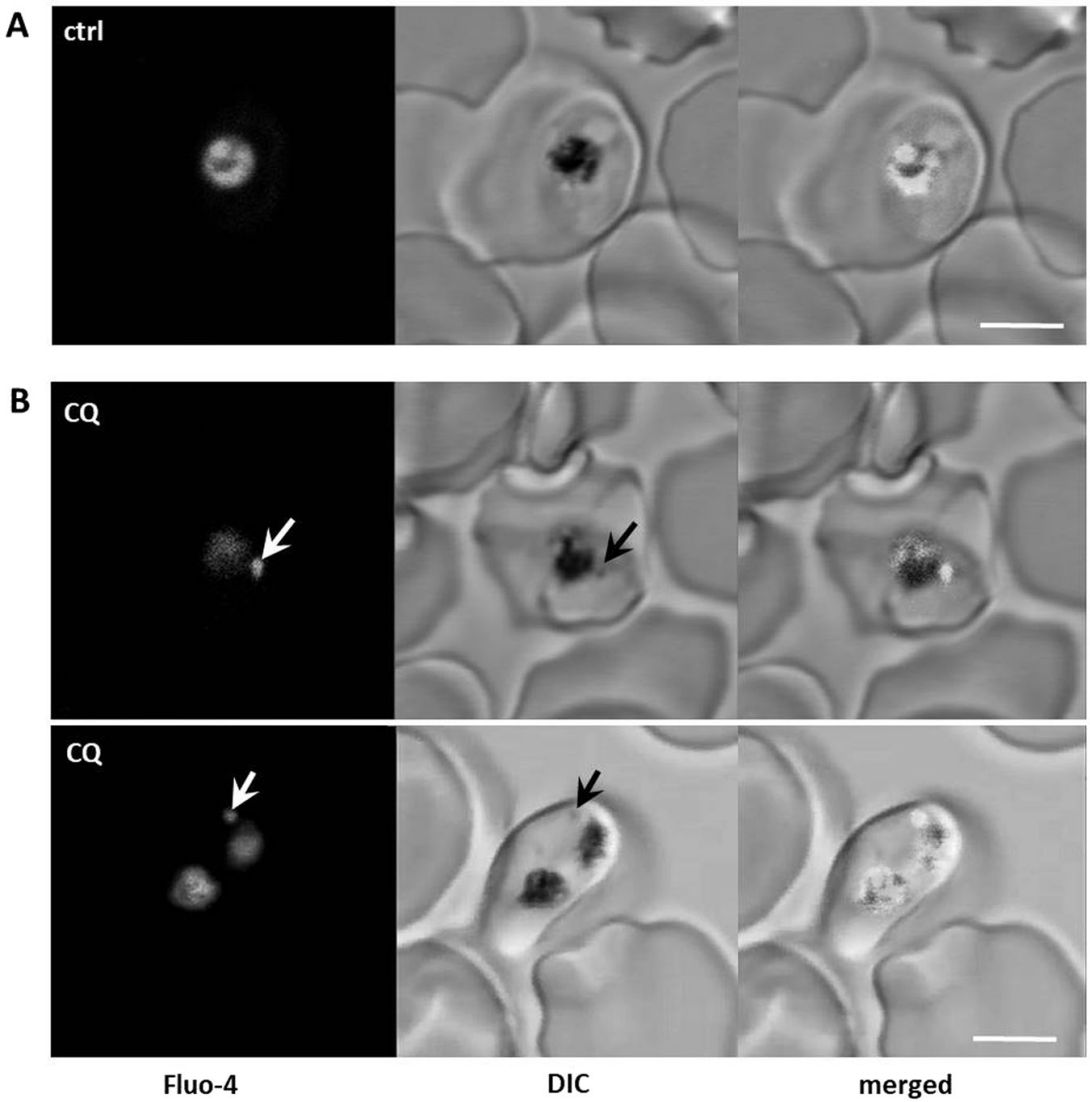
Hz-containing compartments likely originate from the DV. 3D7 parasites were loaded with 5 µM Fluo-4 AM for 1 h, then washed and left untreated (**A**), or incubated with 20x IC_50_ CQ for 2 h (**B**). Fluo-4 remained trapped in the DV of control parasites for the duration of the experiment. CQ-treated parasites showed co-localization of the DV-derived Fluo-4 staining (white arrows) with cytoplasmic hemozoin (black arrows). Scale bars, 5 µm.

To address the origin of the DCS, we pre-loaded 3D7 parasites with Fluo-4 AM before the addition of CQ. We hypothesized that DCS derived from the DV would contain the fluorochrome. Indeed, fluorescently stained compartments containing Hz were seen in the cytosol of 3D7 parasites after CQ exposure (Fig. 5B, Supplementary Video S5), but not in unexposed 3D7 parasites or in Dd2 parasites (exposed or unexposed) (Fig. 5A). These data strongly support the hypothesis that DCS are derived from the DV after exposure of CQS parasites to the drug.

## Discussion

Many studies have attempted to resolve the antimalarial mechanism of action of CQ. How exposure to the drug leads to the death of the parasite and how mutations in PfCRT protect against this effect remain unclear. In this study, we show (i) the appearance of dark cytosolic structures (DCS) containing Hz in the cytosol of CQS but not CQR parasites treated with CQ, (ii) the elimination of DCS in CQS parasites treated with CQ in the presence of VP, and (iii) the delayed onset of irreversibly lethal damage in CQR versus CQS parasites exposed to equitoxic [CQ]_ex_. Together, these and other morphological changes indicate that there is a fundamental difference in the consequences of drug exposure in CQS compared to CQR parasites.

Four hypotheses have been formulated to explain CQ resistance in malaria parasites. The first postulates that the cytosol:DV pH gradient is smaller in CQR than CQS parasites, resulting in reduced CQ influx into the DV and a lower equilibrium [CQ]_DV_ at a given [CQ]_ex_^28,29^. The second hypothesis proposes that the FPIX complex in CQR parasites has reduced affinity for CQ compared to this complex in CQS parasites; therefore, a lower concentration of toxic CQ-FPIX complexes accumulate at a given [CQ]_DV_. The third hypothesis suggests that PfCRT carrying the K76T mutation causes CQ to be transported out of the DV and reduces the amount of CQ in the DV, again lowering the equilibrium [CQ]_DV_ at a given [CQ]_ex_. The fourth hypothesis proposes that CQ may be directly responsible for inhibiting PfCRT function. CQ is thought to bind PfCRT and mutations in PfCRT responsible for CQ resistance (defined as increased CQ IC_50_ values in 48 or 72 h assays) might impede CQ binding by converting binding into efflux events. While the first hypothesis has not been supported by experimental data, multiple studies support the latter three^14,15,30–36^.

There is evidence that CQ resistance cannot be attributed to the K76T amino acid substitution alone. CQR strains incubated with increasing [CQ]_ex_ must accumulate higher internal [CQ] compared to CQS strains to obtain the same efficacy in parasite killing^3,9,16,17,31^. Furthermore, inconsistencies have been found between the presence of the K76T mutation and resistance to CQ *in vitro* and *in vivo*^37–41^. In addition, mutant PfCRT contributes to the shift to the right in the concentration-response curve between CQS and CQR strains, but only accounts for 10–20% of the LD_50_ in CQR parasites^42^. Even at the same [CQ]_DV_, CQR strains are relatively protected from the drug and a second mechanism(s) that prevents CQ from causing lethal effects must be in place^9^. To demonstrate if specific PfCRT polymorphisms are required for the formation of DCS, experiments are now in progress using isogenic parasites that are genetically modified to express wildtype (CQS) or mutant (CQR) forms of PfCRT and exposing these to CQ. A correlation could suggest that CQ drug resistance is not solely concentration-dependent but that PfCRT mutations may also contribute to distinct cellular responses as a consequence of CQ exposure.

Although the discovery of the PfCRT K76T mutation^14,34^ was a milestone in understanding CQ resistance, further studies have revealed that mutant *pfcrt* cannot confer CQR in all genetic backgrounds^22,43^. A link between PfCRT and/or PfMDR1 mutations to antimalarial drug susceptibility has been suggested^44–47^, and additional loci other than *pfcrt* and *pfmdr1* may also contribute to CQ resistance^37^.

Other researchers have proposed that a cytosolic target for CQ accounts for the antimalarial activity^48^. Our results suggest that lethal [CQ]_ex_ affect CQS and CQR parasites differently, supporting the idea that the mechanism of CQ resistance includes factors other than selection of alleles of PfCRT. Reduced affinity of CQ for FPIX, the second hypothesis, is a plausible candidate. Although a shift in affinity curves, as reflected by higher IC_50_ values required for CQR, does not necessarily affect lethality at saturating [CQ]_ex_ in respective CQS or CQR strains, it may still alter the kinetics of parasite elimination, as indeed shown for the protracted lethality in CQR over CQS.

Increased survival in the presence of equitoxic [CQ]_ex_ of CQR compared to CQS parasites was revealed by incubating trophozoites in CQ for varying lengths of time. Even when exposed to [CQ]_ex_ corresponding to equivalent IC_50_ values, CQR parasites were viable longer than CQS parasites. It has been suggested that equal internal [CQ] alone are not sufficient to eliminate CQR parasites compared to CQS parasites^17^. Our results support the possibility that a regulatory mechanism found in CQR parasites reduces the time course of toxicity of CQ in CQR, albeit having the same steady-state endpoint effect, i.e. complete elimination (Fig. 1). Our data suggest that CQR parasites not only reduce CQ accumulation in the DV, but may also delay the initiation and progression of cell death. Additionally to the aforementioned effect, our data also show that the cellular damage elicited by such exposures also differs between CQR and CQS strains.

In human cells, CQ is known to induce programmed cell death (PCD) through both apoptosis and autophagy pathways^49^. Recent investigations of cell death pathways in protozoan parasites have found evidence of PCD^50^. The possibility of CQ-induced apoptotic cell death in malaria parasites is unlikely since *Plasmodium* spp. lack caspases, a central component of apoptosis. Other classical apoptotic features, such as chromatin condensation, DNA fragmentation, disruption of the plasma membrane and nucleus dissolution, were also not observed in CQ-treated parasites^51–53^. In contrast, CQ-induced autophagic-like PCD has been suggested in malaria parasites^51,54^. Although autophagy is a strategic mechanism for cell survival, it can also lead to the death of an organism. Autophagic vacole formation after CQ exposure in malaria parasites was noted in the 1970s^55^. Cytoplasmic vacuolization similar to autophagy was confirmed by EM^51^. These findings are supported by our EM experiments, which revealed morphological effects in CQ-treated CQS parasites that included the accumulation of membranes in the DV and the formation of large vacuoles in the cytoplasm. The accumulation of undigested and unprocessed autophagic vesicles may be caused by defective lysosomal proteolysis or hyperactive autophagy induction^56^. Both possibilities seem plausible in CQ-exposed parasites.

Although some researchers have addressed the biology of cell death after CQ exposure in *P*. *falciparum*^24,51,57,58^, a major impediment to such studies is the lack of molecular tools available to study cell death in malaria parasites, e.g., antibodies against regulatory proteins to such as LC3 or caspase-3 that are typically involved in the initiation of PCD. LD_50_-directed quantitative trait loci analysis in CQS versus CQR strains revealed differences in loci that encode proteins linked to autophagy pathways, such as the autophagy-related genes (ATG)^42^, but no genes that are involved in apoptosis^42,54,58,59^. Further work in this area may lead to a better understanding of the antimalarial action of CQ and may reveal additional resistance mechanisms in CQR parasites.

While some PCD-related features have been described in CQ-exposed parasites through analysis of EM images^24,51,60^, no reports describe the appearance of DCS within the cytosol of CQS - and not CQR - parasites after exposure to the drug. The appearance of compartmentalized Hz crystals in our EM images correlated with the DCS observed in DIC images. The CQ-induced redistribution of Hz, or FPIX, from the DV to the parasite cytosol has been previously shown using TEM^61^. However, the effect of CQ on elevated levels of FPIX in the cytosol was only investigated in the CQS 3D7 strain and no comparisons were made between CQS and CQR strains. These findings supported previous work by Ginsburg and coworkers, who hypothesize that the principal toxic effect of CQ is manifest outside the DV and is linked to the binding of CQ to cytosolic FPIX^62^. Free FPIX is thought to easily escape the DV and is rapidly degraded by GSH. As much as 70% of the FPIX generated through hemoglobin digestion was found to be detoxified by cytosolic glutathione^48^. Although the presence of iron in the cytosol has previously been shown, the compartmentalization of Hz in DCS in CQS but not CQR parasites has not been previously described.

Thus, exposure of CQS parasites to lethal [CQ]_ex_ led to the appearance of multiple morphological changes prior to the leakage of DV contents into the cytosol. These changes were not observed in CQR parasites exposed to equally lethal [CQ]_ex_, suggesting that the consequences of equipotent CQ exposure differ between them within the first 3 h of exposure.

The increase in Hz-containing DCS in the parasite cytosol is not due to rupture of the DV membrane. Experiments with Fluo-4 AM, which is actively transported into the DV by PfMDR1^26^, showed that the DV membrane remained intact when the parasite was exposed to [CQ]_ex_ that cause irreversibly lethal damage, as there was no major leakage of the fluorochrome into the cytosol at a time when the parasites were destined to die. However, fluorescent staining of Hz-containing DCS in the cytosol is most plausible if the compartments formed at the DV and contained DV contents, including Fluo-4. The localization of Fluo-4 fluorescence in DCS provides strong evidence that these structures arose from the DV.

Clearly, further studies are needed to elucidate the implications of the presence of DCS in CQS parasites after CQ exposure and their absence from CQR parasite strains. Understanding PCD in *P*. *falciparum* and obtaining insight into how CQR parasites maintain homeostasis even under drug pressure may reveal a unique mechanism in CQR parasites that could be exploited to potentially reverse CQ resistance, opening new possibilities to restore the clinical utility of this drug.

## Material and Methods

### Parasite strains and culture conditions

CQS (3D7, HB3) and CQR (FCB, Dd2) parasite strains were continuously cultured as described by Trager and Jensen^63^, with modifications. Briefly, parasites at 5% hematocrit were propagated in RPMI 1640 (Life Technologies, Burlington, ON, Canada) supplemented with 25 mM HEPES, 2 mM L-glutamine, gentamicin (20 µg/ml) (Life Technologies, Burlington, ON, Canada), 100 µM hypoxanthine (Sigma-Aldrich, Oakville, ON, Canada), and 0.5% AlbuMAX I (Life Technologies, Burlington, ON, Canada). Parasites were maintained at 37 °C under an atmosphere of 5% CO_2_, 3% O_2_ and 92% N_2_. A^+^ red blood cells were obtained from the Interstate Blood Bank (Memphis, TN, USA). Giemsa (Sigma-Aldrich, Oakville, ON, Canada)-stained blood smears were prepared daily to monitor parasite growth. For synchronization, parasites were exposed to 5% D-sorbitol (BioShop Canada, Burlington, ON, Canada) for 10 min at 37 °C; sorbitol was removed and parasites were washed once before placed back into culture. To obtain highly synchronous parasite cultures, this treatment was repeated after 6 h.

### Growth inhibition assays

CQ was obtained from Sigma-Aldrich Canada (Oakville, ON, Canada) and dissolved in dH_2_O. Growth inhibition assays were performed as described^19^, with modifications. Highly synchronized ring stage parasites were diluted to a final parasitemia of 0.5% and a hematocrit of 2%. A total of 100 µl culture medium per well was prepared in a 96-well plate assay, with a drug dilution series of 1:3, ranging from 1 µM to 0.15 nM. Plates were incubated at 37 °C, 5% CO_2_, 3% O_2_ and 92% N_2_ for 72 h, then frozen and stored at −80 °C. Plates were thawed at room temperature and 100 µl 2x lysis buffer (20 mM Tris pH 7.5, 5 mM EDTA, 0.008% saponin, 0.08% Triton X-100, 0.2 µl SYBR Green I/ml) (SYBR Green I: Sigma Aldrich, Oakville, ON, Canada; all other chemicals: BioShop Canada, Burlington, ON, Canada) was added to each well. Plates were incubated in the dark for at least 1 h. Fluorescence intensity was determined using a Synergy H4 plate reader (Fisher Scientific, Nepean, ON, Canada) with 485 nm excitation and 520 nm emission wavelengths. IC_50_ values were calculated by fitting concentration response curves with a custom procedure for IGOR Pro 6.2 based on an R script kindly provided by Le Nagard and used as described^18,64^.

### Chloroquine exposure assay

Wells containing highly synchronized early trophozoite stage parasites were prepared in triplicate at 1% parasitemia and 2% hematocrit in 100 µl culture medium in a 96-well plate. Parasites were either left untreated (control) or exposed to [CQ]_ex_ of 10x, 20x or 30x their IC_50_ values (Table 1) and incubated for various lengths of time. After incubation, parasites were washed with culture medium to remove CQ and resuspended in 100 µl fresh culture medium. Parasites were incubated for 48 h (length of time for complete assay) at 37 °C, 5% CO_2_, 3% O_2_ and 92% N_2_, after which the plates were frozen at −80 °C. Analysis was performed with SYBR Green I detection as described for the growth inhibition assay.

### Live cell imaging

For DCS counts in CQS and CQR parasites, synchronized trophozoite stage parasites were exposed to 2x or 10x their CQ IC_50_, or left unexposed (control). A constant temperature of 37 °C was maintained during imaging using a stage-top incubator (Tokai Hit, Shizuoka-ken, Japan). Infected erythrocytes were thoroughly screened for presence or absence of DCS and grouped in time intervals of 30 min. During each time interval, at least 101 (average 169) parasites were examined. DIC images were taken with a Zeiss LSM 710 confocal microscope equipped with a water-corrected objective (C-apochromat 63x/1.20 W Korr M27) and 488 nm laser (12.5 mW, 3.0%) and analyzed using the ZEN 2010 software (Carl Zeiss MicroImaging, Oberkochen, Germany).

For analysis of DV membrane integrity, synchronized early trophozoite stage 3D7 and Dd2 parasites were loaded with 5 µM Fluo-4 AM (Life Technologies, Burlington, ON, Canada) in Ringer’s solution (122.5 mM NaCl, 5.4 mM KCl, 1.2 mM CaCl_2_, 0.8 mM MgCl_2_, 11 mM D-glucose, 10 mM HEPES, 1 mM NaH_2_PO_4_, pH 7.4) for 50 min at 37 °C to allow Fluo-4 accumulation in the DV. Parasites were then washed twice with Ringer’s solution to remove excess fluorochrome and left untreated, or incubated with 10 µM CQ (400x 3D7 IC_50_). For analysis, parasites were transferred onto poly-L-lysine (Sigma-Aldrich, Oakville, ON, Canada) coated cover slips in a microscope chamber with or without 10 µM CQ in Ringer’s solution. Parasites were kept at 37 °C using a stage-top incubator (Tokai Hit, Shizuoka-ken, Japan) for the entire experiment. Ringer’s solution was replaced every 60 min with or without 10 µM CQ for treated and control groups, respectively. To eliminate the possibility of reduced fluorescence in the DV and increased fluorescence in the cytosol due to lengthy exposure time^65^, the parasites were kept in the dark at 37 °C and single images were taken every 15 min with low laser intensity (488 nm laser; 12.5 mW; 0.8% laser power). Parasites were imaged using a time series function on a Zeiss LSM710 confocal microscope (Carl Zeiss, Oberkochen, Germany) equipped with a water-corrected objective (C-apochromat 63x/1.20 W Korr M27) and 488 nm laser (12.5 mW, 0.8%). Images were analyzed using ImageJ 1.47q (National Institutes of Health, USA).

### Electron microscopy

CQS and CQR parasites were exposed to 20x IC_50_ CQ or left unexposed (control) for 3 h, fixed and prepared for electron microscopy. Parasite enrichment was performed using synchronized trophozoite stage parasites. Cultures at 5% parasitemia were used for Easycoll (Cedarlane Laboratories, Burlington, ON, Canada) enrichment using a density gradient. In a 15 ml tube, a gradient of 4 ml of 80% Easycoll, 3 ml of 70% Easycoll, 3 ml of 60% Easycoll and 3 ml of 40% Easycoll was prepared. The Easycoll gradient was adjusted using warm Ringer’s solution. Approx. 500 µl parasite culture was resuspended in 2 ml Ringer’s solution and layered on top of the gradient. Tubes were centrifuged at 4,700x g (Thermo Fisher Scientific, Mississauga, ON, Canada) for 45 min at 24 °C with low acceleration and decelerated without brake. Trophozoite stage parasites accumulated at the interphase between 60% and 70% Easycoll. Parasites isolated from this phase were washed twice with Ringer’s solution and were typically enriched to 62–93% parasitemia. The enriched trophozoite pellet was resuspended in 500 µl fixation solution containing 50 mM cacodylic acid (Carl Roth, Karlsruhe, Germany), 1% paraformaldehyde (Applichem, Darmstadt, Germany), 2.5% glutaraldehyde (Merck KGaA, Darmstadt, Germany), 2.5 mM MgCl_2_ and 50 mM KCl, pH 7.2. Samples were centrifuged at 1,700 x g for 20 min at 4 °C, the supernatant aspirated and another 500 µl fixation solution was added overnight at 4 °C. Fixation solution was removed and the pellet was washed three times with 50 mM cacodylic acid, pH 7.2. Samples were stained with 2% OsO_4_ in 50 mM cacodylic acid, pH 7.2 at 4 °C for 2 h, then washed with H_2_O, stained with 0.5% aqueous uranyl acetate at 4 °C overnight and washed with H_2_O. Samples were dehydrated and embedded in epon 812 (Serva, Heidelberg, Germany). Ultrathin sections of 50–70 nm thickness were analyzed using a JEM-2100 TEM (JEOL, Eching, Germany) at 200 kV. Images were taken using a 4 K camera from Tietz Video and Image Processing Systems (TVIPS, Gauting, Germany).

## Electronic supplementary material

Supplementary Material

Supplementary Video S1

Supplementary Video S2

Supplementary Video S3

Supplementary Video S4

Supplementary Video S5
