# Supplementary material for: Chloroquine exposure triggers distinct cellular responses in sensitive versus resistant Plasmodium falciparum parasites
Source: Sci Rep. 2018 Jul 24;8:11137. doi: 10.1038/s41598-018-29422-6 (PMC6057915; doi:10.1038/s41598-018-29422-6)
Supplement: Supplementary file 1 — Supplementary Material [file 41598_2018_29422_MOESM1_ESM.pdf]

Supplementary Information:

Chloroquine exposure triggers distinct cellular responses in sensitive versus resistant *Plasmodium falciparum* parasites

*S.J. Reiling[1], G. Krohne[2], Oliver Friedrich[3], Timothy G. Geary[1], and P. Rohrbach[1,]**

[1] Institute of Parasitology, McGill University, Ste. Anne de Bellevue (Montréal), Québec, Canada

[2] Division of Electron Microscopy, Biocenter, University of Würzburg, Germany

[3] Institute of Medical Biotechnology, Friedrich-Alexander University of Erlangen-Nürnberg, Germany

* corresponding author:

Institute of Parasitology, McGill University, 21111 Lakeshore Road, Ste. Anne de Bellevue, QC H9X-3V9, Canada

Tel.: (514) 398-7726

email: petra.rohrbach@mcgill.ca

Supplementary Figures:

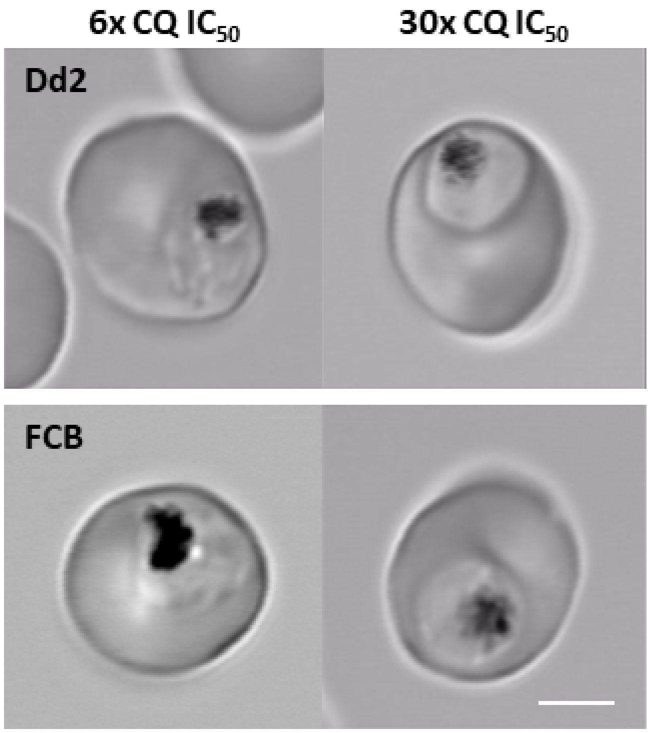

Figure S1: No formation of cytosolic Hz-containing compartments in CQR parasites. CQR parasite strains Dd2 and FCB were incubated with 6x IC_{50} or 30x IC_{50} CQ for 4 h. No DCS could be observed. Experiments were done in triplicates on independent days. Scale bar, 5 µm.

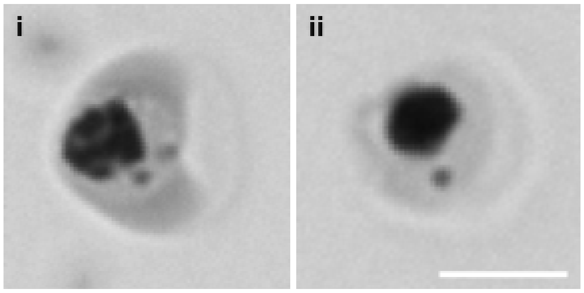

Figure S2: Hz-containing compartments are visible in methanol-fixed unstained parasites. 3D7 parasites were incubated with their 20x IC_{50} CQ concentration for 3 h, then air-dried and fixed with methanol. DCS were easily observed in the parasite cytosol. i-ii, representative images. Scale bar, 5 µm.

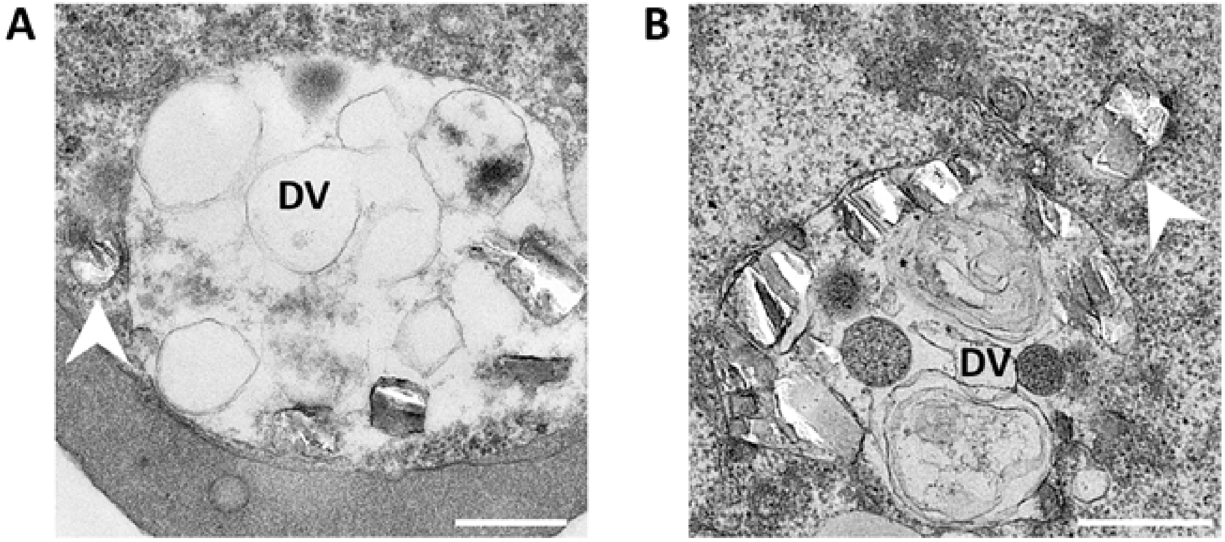

Figure S3: Sample preparation for EM imaging. 3D7 parasites were incubated with their 20x IC_{50} CQ concentration for 3 h and then either left non-enriched (**A**) or enriched using a Percoll gradient (**B**). Hz-containing compartments (white arrowheads) were detected in the parasite cytosol for both preparation techniques. DV, digestive vacuole. Scale bars, 500 nm.

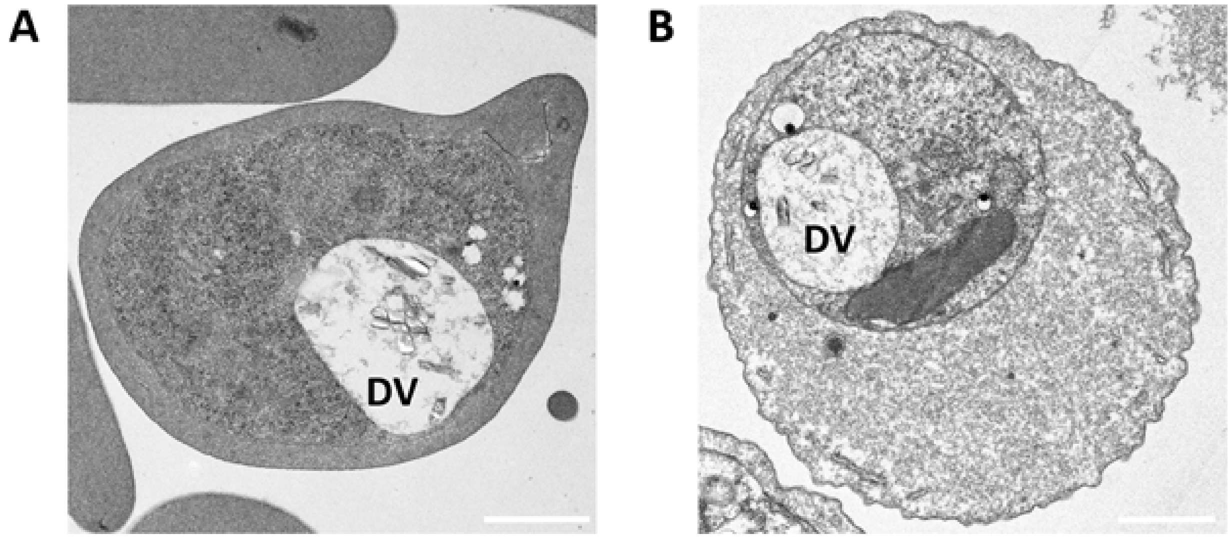

Figure S4: Untreated parasites showed no cytosolic Hz-containing compartments. 3D7 (**A**) and Dd2 (**B**) parasites were prepared for EM without CQ exposure (controls). DV, digestive vacuole. Scale bars, 1 µm.

Supplementary Videos:

Video S1: Dd2 parasite, incubated in Ringer's solution for 3 h at 37°C (control).

Video S2: Dd2 parasite, incubated with 500 nM CQ in Ringer's solution for 3 h at 37°C.

Video S3: 3D7 parasite, incubated in Ringer's solution for 3 h at 37°C (control).

Video S4: 3D7 parasite, incubated with 500 nM CQ in Ringer's solution for 3 h at 37°C.

Video S5: 3D7 parasites, pre-incubated with Fluo-4 AM for 1 h, then treated with 20x CQ IC_{50} for 2 h to induce formation of Hz-containing compartments. Fluorescence staining of the Hz-containing compartments indicates that the DCS must have originated from the DV, where Fluo-4 accumulates.
